# Intratumor IL-17-Positive Mast Cells Are the Major Source of the IL-17 That Is Predictive of Survival in Gastric Cancer Patients

**DOI:** 10.1371/journal.pone.0106834

**Published:** 2014-09-08

**Authors:** Xiaosun Liu, Hailong Jin, Geer Zhang, Xianke Lin, Chao Chen, Jianyi Sun, Yu Zhang, Qing Zhang, Jiren Yu

**Affiliations:** Department of Gastrointestinal Surgery, the First Affiliated Hospital, Medical College, Zhejiang University, Hangzhou, China; The University of Hong Kong, China

## Abstract

Interleukin-17 (IL-17) is prevalent in tumor tissue and suppresses effective anti-tumor immune responses. However, the source of the increased tumor-infiltrating IL-17 and its contribution to tumor progression in human gastric cancer remain poorly understood. In this study, we enrolled 112 gastric cancer patients, immunofluorescence was used to evaluate the colocalization of CD3, CD4, CD56, CD20, CD68, and mast cell tryptase (MCT) with IL-17. Immunohistochemistry was used to evaluate the distribution of microvessel density (CD34), CD66b^+^, CD68^+^, and FoxP3^+^ cells in different microanatomical areas. Prognostic value was determined by Kaplan-Meier analysis and a Cox regression model. The results showed that mast cells, but not T cells or macrophages, were the predominant cell type producing IL-17 in gastric cancer. Significant positive correlations were detected between densities of mast cell-derived IL-17 and microvessels, neutrophils, and regulatory T cells (Tregs). Futhermore, we found that the majority of vascular endothelial cells expressing Interleukin-17 receptor (IL-17R). Kaplan-Meier analysis revealed that increasing intratumor infiltrated mast cells and IL-17^+^ cells, as well as MCT^+^ IL-17^+^ cells, were significantly associated with worse overall survival. These findings indicated that mast cells were the major source of IL-17 in gastric cancer, and intratumor IL-17 infiltration may have promoted tumor progression by enhancing angiogenesis in the tumor microenvironment through the axis of IL-17/IL-17R. IL-17-positive mast cells showed a prognostic factor in gastric cancer, indicating that immunotherapy targeting mast cells might be an effective strategy to control intratumor IL-17 infiltration, and consequently reverse immunosuppression in the tumor microenvironment, facilitating cancer immunotherapy.

## Introduction

During recent decades, increasing attention has been paid to the mechanism(s) of escaping immunosurveillance in the tumor microenvironment and, especially, the relationship between inflammation and immunosuppression. One of the most important components of the immune system in inflammation associated with cancer has recently been recognized as interleukin-17 (IL-17). IL-17 has pleiotropic functions and multiple targets that have mostly been explored in mouse models, but increasingly linked to human diseases [1]–[5]. IL-17 has been identified in various tumors, including breast cancer [6], gastric cancer [7], [8], colorectal cancer [9], carcinogen-induced skin cancer [10], intrahepatic cholangiocarcinoma [11], and hepatocellular carcinoma [12]. IL-17-producing cells gradually increase in number in the tumor microenvironment during tumor development and are correlated with poor survival in cancer-related patients.

It has long been considered that the major cellular source of IL-17 is CD4^+^ T lymphocytes (Th17 cells). Recently, many studies targeting IL-17 have demonstrated that IL-17 production is not restricted to Th17 lymphocytes, but is also found in a variety of adaptive and innate immune cells, including γδT cells, NKT cells, NK cells, mast cells, and granulocytes [3], [13].

Gastric cancer is one of the most common cancer types and a leading cause of cancer-related death worldwide [14]. More than 1 million new patients are diagnosed with gastric cancer every year worldwide, 42.5% of whom are in China. Traditional therapeutic strategies, including surgery, chemotherapy and radiotherapy are the main treatments for gastric cancer. Recently, immunotherapy of malignancies has been brought into focus [15]–[17]. However, tumor-associated immunosuppression is a major challenge for immunotherapy, because it weakens the cytotoxic activity of effective T cells and natural killer cells. During tumor development, tumor immunosuppression is commonly associated with excessive and uncontrolled inflammation in the tumor microenvironment [18]. It is well-known that multiple inflammatory cells infiltrate tumors, including mast cells, macrophages subtypes, neutrophils, as well as T and B lymphocytes, a hallmark of cancer-associated inflammation [19].

In the current study, we examined the cellular sources of IL-17, distribution, functional relevance, and predictive value of IL-17-producing cells in 112 patients with gastric cancer. We provide novel insights into the potential mechanism(s) of IL-17 in the tumor microenvironment in patients with gastric cancer by evaluating the relationship between IL-17-producing cells and inflammatory mediators. Our aim is to provide more information that may be useful for designing more effective cancer immunotherapies that target IL-17.

## Materials and Methods

### Ethics statement

Prior to the research, informed consent was obtained from each patient. Appropriate permission was granted by the ethics committee of the First Affiliated Hospital, Medical College, Zhejiang University.

### Patient samples

In total, 112 patients between February 2009 and March 2010 in our hospital were enrolled. Patients who met the following criteria were selected: a) diagnosed as gastric cancer based on pathology; b) received effective resection. The exclusion criteria were patients who had evidence of distant metastasis, evidence of concurrent autoimmune disease, HIV, or syphilis and patients who received anticancer therapy before surgery. The criteria of effective resection were defined as resection with no residual tumor (R0) or microscopic residual tumor (R1) according to the 7^th^ edition of the American Joint of Committee on Cancer. After curative resection, patients received adjuvant chemotherapy based on 5-flurouracil and platinum according to the pathological TNM classification. Patients were followed-up with 3-6 months interval, including assays of their peripheral blood for tumor markers and ultrasonography, and enhanced CT every 6 months, endoscope every 12 months. Clinical characteristics of patients are shown in Table 1.

**Table 1:** Association between intratumor MCT/IL-17 double-positive cells and clinicopathological characteristics.

Variables		Number of Intratumor MCT/IL-17 double positive cells		
	N0. (%)	Low	High	P-value
*Gender*				0.872
Male	78(69.6)	38	40	
Female	34(30.4)	16	18	
*Age*				0.039
* ≤*60	59(52.7)	23	36	
>60	53(47.3)	31	22	
*HP status*				0.800
Negative	65(58.0)	32	33	
Positive	47(42.0)	22	25	
*Tumor size*				0.559
* ≤*5 cm	93(83.0)	46	47	
>5 cm	19(17.0)	8	11	
*Tumor location*				0.874
Higher	13(11.6)	6	7	
Lower+middle+others	99(88.4)	48	51	
*Degree of differentiation*				0.381
Well and moderately	27(24.1)	15	12	
Poorly	85(75.9)	39	46	
*T stage* a				
Tis+T1+T2	36(32.1)	20	16	0.285
T4	76(67.9)	34	42	
*Lymph node metastasis* a				0.651
N0	33(29.5)	17	16	
N1+N2+N3	79(70.5)	37	42	
*TNM stage* a				0.600
Tis+I+II	49(43.7)	25	24	
III	63(56.3)	29	34	

a 7^th^ Edition of American Joint of Committee On Cancer.

All data was analyzed by Chi-square test.

### Immunohistochemistry and Immunofluorescence

Routine hematoxylin and eosin staining was performed to confirm the pathological diagnosis, intratumor region was defined as less than 2 cm bordering the margin of the stomach tumor, while normal region was defined as at least 5 cm bordering the margin of the stomach tumor.

### Immunohistochemistry

Immunohistochemistry was performed as described in our previous study [20]. Primary antibodies were goat anti-IL-17 (R&D Systems, UK), mouse anti-CD66b (BD Pharmingen, USA), mouse anti-CD68 (Abcam, USA), mouse anti-FoxP3 (Abcam, USA), and mouse anti-CD34 (Beijing Zhongshan Golden Bridge Biotech, China). For the negative control, the primary antibody was replaced with phosphate-buffered saline (PBS).

### Immunofluorescence

For immunofluorescence, similar methods were used, except that endogenous peroxidase activity was not blocked and the primary Ab used was an antibody cocktail. Goat anti-IL-17 and goat anti-IL-17R (both from R&D Systems, USA) were used to detected IL-17^+^ cells and IL-17R expression. A panel of antibodies reactive with CD3, CD20, CD34, CD56, c-kit (mouse monoclonal, all from Beijing Zhongshan Golden Bridge Biotech, China), CD4 (mouse monoclonal, Leica, German), CD66 (mouse monoclonal, BD Pharmingen, USA), CD68, FoxP3 and mast cell tryptase (MCT) (mouse monoclonal, all from Abcam, USA) were used. After incubation overnight at 4°C, sections underwent incubation with a mixture of secondary antibodies: Alexa Fluor 488/568-conjugated donkey anti-mouse or Cy3/Alexa Fluor 488-conjugated donkey anti-goat or Alexa Fluor 488 donkey anti-rabbit (all from Invitrogen, USA) at 37°C for 1 h. Slides were mounted with Vectashield containing DAPI (Vector Laboratories, USA) and visualized using a fluorescence imaging microscope (Olympus BX51, Japan) coupled to a CCD camera (Nikon DS-Ri1). The images were analyzed using the NIS-ELements BR 3.2 software.

Negative controls, in which PBS was used in place of the primary antibodies, were included for each marker.

### Quantifying immunostaining parameters

Data were obtained by manually counting positively stained cells in 10 representative fields of normal and intratumoral regions under 400× high-power magnification. Specifically, vascular endothelial cells or clusters of brown-stained cells were identified as microvessels only if they had clear boundaries with adjacent structures [21]. Densities were determined by computing the mean number of positively stained cells per high power microscopic field (HPF). Double staining was determined by manual counting of positive cells in 10 high-power fields; the proportions of IL-17-expressing cells per surface marker were calculated. All analyses were performed by two independent, blinded observers (GE Z and XK L), and the average counts of the two observers were used in the following analysis.

### Statistical analysis

The median values of CD34^+^, CD66b^+^, CD68^+^, FoxP3^+^, and mast cell tryptase (MCT^+^) and IL-17^+^ cells were used as cut-offs to define the subgroups in our results. Student's t-tests were used to compare infiltration of different immune cell subsets into tumor tissue and corresponding normal tissue. Chi-squared test was used to assess the relationship between IL-17-producing cells and clinicopathological features. Correlations between microvessel density (CD34), neutrophils (CD66b), macrophages (CD68), regulatory T cells (FoxP3), and IL-17-producing cells were assessed using Pearson's or Spearman's correlation coefficient. Overall survival was defined as the interval between date of surgery and date of death or last follow-up, whichever occurred earlier. Survival function estimates were computed using the Kaplan-Meier method. A Cox proportional hazards model was used to assess the effect of IL-17-producing cells on overall survival. A two-sided p value < 0.05 was considered to indicate statistical significance. All statistical analyses were performed using GraphPad Prism (ver. 5.00 for Windows; GraphPad Software, San Diego, CA, USA) and the SPSS (ver. 16.0; SPSS Inc., Chicago, IL, USA) software.

## Results

### Study population

Patients' characteristics are shown in Table 1. In total, 78 males and 34 females were enrolled. The age of the study population ranged from 33 to 89 (median 60) years. Of the 112 gastric cancer patients, H. pylori infection status was positive for 65 patients, while 47 was negative. 110 patients received R0 resection and 2 patients received R1 resection. Pathological TNM classifications were based on the 7^th^ edition of the American Joint of Committee on Cancer: 33 patients had no lymph node metastasis, while 21 were N1, 22 were N2, and 36 were N3. 1 patient belonged to Tis, 23 patients belonged to stage I, and 25, 63 patients to stages II, III, respectively. 3 (2.7%) patients lost to follow up and 39 (34.8%) patients died during the observation period. The median follow up time was 51 months, ranging from 39 to 57 months and the overall survival (OS) rates were 83.0% at 1 year, 71.4% at 3 years and 65.7% at 4 years, respectively.

### IL-17 was expressed predominantly by MCT^+^ mast cells

**Figure 1:**
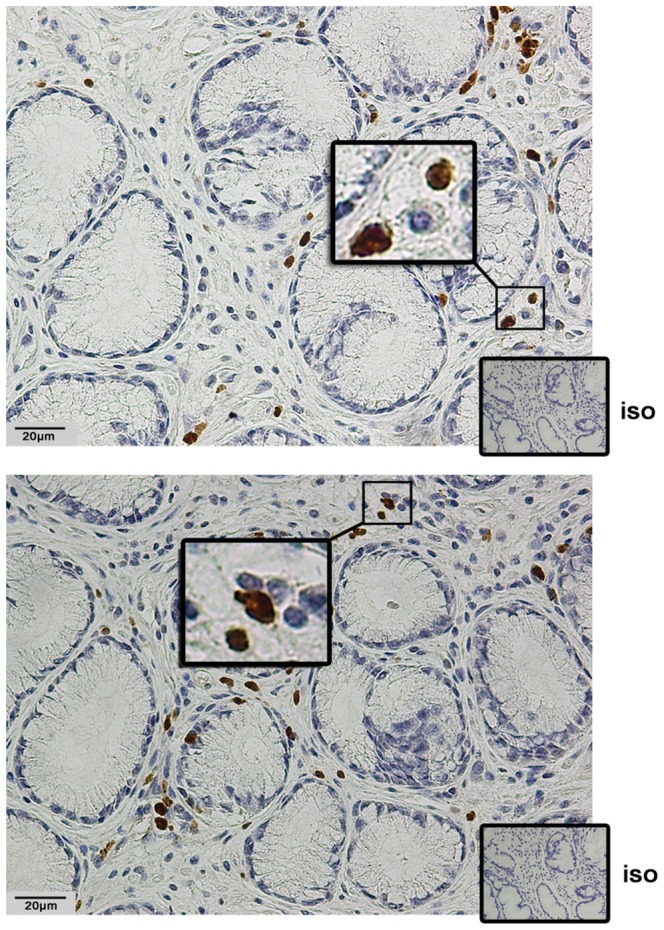
Immunostaining for IL-17 in gastric cancer show different cell morphology. Squares indicate different examples (brown, shown at 400× magnification).

**Figure 2:**
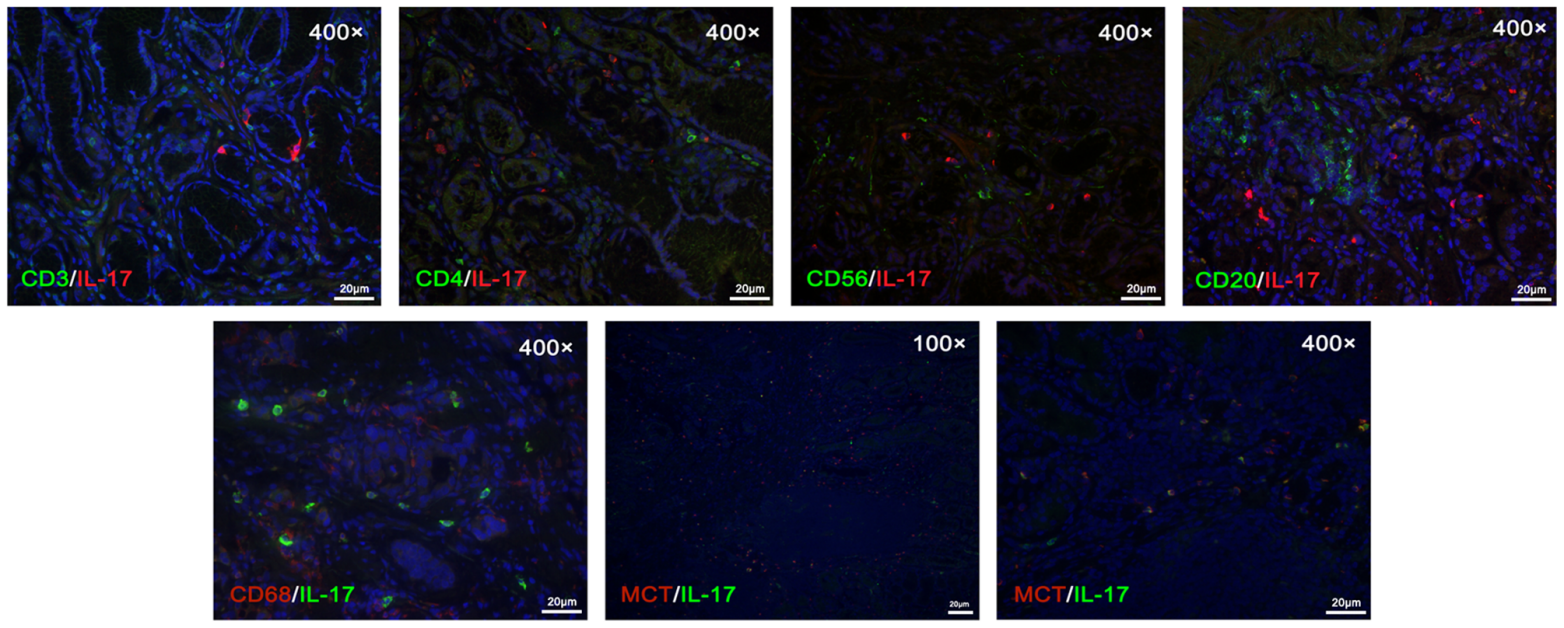
Gastric cancer tissue samples (n = 20) were stained for IL-17 (red and green) and indicated markers. Sections were counterstained with DAPI. Markers of CD3 or CD4 (green) and B cell marker CD20 (green), nature killer (NK) marker CD56 (green) and macrophage marker CD68 (orange), as well as mast cell tryptase (MCT) (orange) are shown at 400× magnifications.

Random samples of 20 patients from the entire cohort were used to evaluate the distribution and phenotype of IL-17-producing cells in intratumor region and the corresponding normal tissues (Fig.1). We found cytoplasmic IL-17 staining in cells with an ovoid phenotype, as described previously [13], and in cells of a more irregular shape. Furthermore, we found that the IL-17-expressing cells were more frequent in intratumor tissues than normal tissues. To identify the IL-17^+^ cells in intratumor tissues, we performed colocalization experiments and calculated the proportion of IL-17^+^ cells in each subset. Firstly, we performed colocalization of c-kit, an additional mast cell marker, with mast cell tryptase (MCT), the results showed that all MCT^+^ cells expressing c-kit (Fig. S1), indicating that MCT could be used to identified mast cells representatively. In cells of the ovoid phenotype, we selected a number of T cell, B cell, and NK cell markers: specifically, CD3, CD4, CD20, and CD56, and assessed their colocalization with IL-17. However, CD20^+^ IL-17^+^ and CD56^+^ IL-17^+^ cells were identified, albeit rarely (< 1%; Fig.2, upper right panels). Although occasional CD3^+^ IL-17^+^ and CD4^+^ IL-17^+^ cells were observed, the mean percentages of IL-17^+^ cells in both subsets were 12.4% (2.2-34.1%) and 8.4% (0–26.7%; Fig.2, upper left panels; Table 2) We next investigated the phenotype of IL-17^+^ cells of irregular shape; macrophages have been suggested as potential sources of IL-17 production [22]. Consistent with this, up to 18.5% of the IL-17-producing cells in gastric cancer tissues were macrophages (Fig.2, lower left panel; Table 2). However, the majority of IL-17-producing cells remained unidentified. To determine whether mast cells were an additional source of IL-17 production in gastric cancer, we performed colocalization of IL-17 and mast cell tryptase (MCT). We found that the majority of IL-17^+^ cells colocalized with MCT^+^ cells (40–60% of IL-17^+^ cells; Fig.2, lower right panel; Table 2).

**Table 2:** Intratumor percentage of double-positive cells compared with IL-17^+^ cells.

		Cells of ten fields	Percentage of IL-17^+^ cells (%)	Percentage of IL-17^+^ cells (%)
(N = 20)		(Mean)	(Mean)	(Minimum/Maximum)
CD3 analysis	CD3^−^IL-17^+^	55		
	CD3^+^IL-17^+^	7.8	12.4	2.2/34.1
CD4 analysis	CD4^−^IL-17^+^	49		
	CD4^+^IL-17^+^	4.5	8.4	0/26.7
CD68 analysis	CD68^−^IL-17^+^	45		
	CD68^+^IL-17^+^	3.2	6.6	0/18.5
MCT analysis	MCT^−^IL-17^+^	36.3		
	MCT^+^IL-17^+^	49.5	57.2	40/60

Cell numbers were assessed in ten independent fields at 400× magnification. Average cell numbers (third column) and ratios to the total amount of IL-17^+^ cells (fourth column) for CD3^+^IL-17^+^ (n = 20), CD4^+^IL-17^+^ (n = 20), CD68^+^IL-17^+^ (n = 20), MCT^+^IL-17^+^ (n = 20) cells were shown.

### Distribution of MCT^+^IL-17^+^ cells and correlation with clinicopathological features

**Figure 3:**
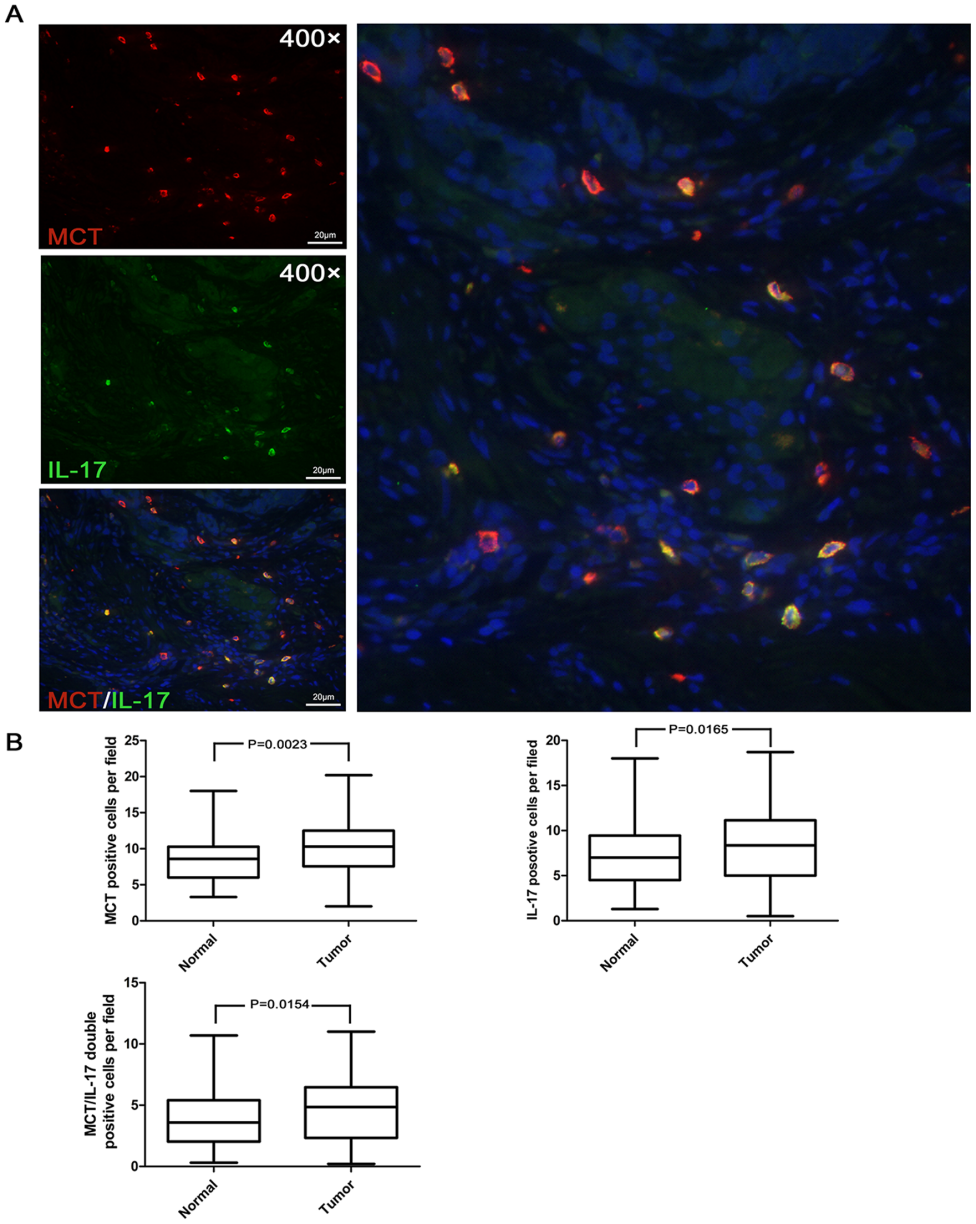
The prevalence of MCT^+^ cells (orange) and IL-17^+^ cells (green) in 112 paired gastric cancer tissue samples. A. Mast cells highly express IL-17 in gastric cancer (merged in yellow, right panel). B. MCT^+^ cells and IL-17^+^ cells, as well as MCT^+^IL-17^+^ cells in intratumor tissues were significant higher than those of corresponding normal tissues.

To confirm that mast-cell-derived IL-17 was a major cellular source of IL-17 in the gastric cancer microenvironment, we performed immunofluorescence double-staining of intratumor and normal sample tissues from the 112 patients. The majority of IL-17^+^ cells double-stained strongly with MCT (14–68.1% of IL-17 expressing cells) in intratumor tissues (Fig. 3A). We found that the densities of MCT^+^ mast cells and IL-17^+^ cells in intratumor tissues were found to be significantly higher than in normal tissues (MCT^+^_T_ vs. MCT^+^_N_, 10.02±4.06 vs. 8.66±3.29, p = 0.0023; IL-17^+^_T_ vs. IL-17^+^_N_, 8.16±3.89 vs. 7.18±3.48, p = 0.0165; Fig. 3B). Moreover, the density of MCT^+^ IL-17^+^ cells in intratumor tissues was significantly higher than that in normal tissues (4.55±2.48 vs. 3.93±2.24, p = 0.0154; Fig. 3B).

To identify significant associations between clinicopathological features and MCT^+^ IL-17^+^ cells, the chi-squared test or fisher's exact test was used (shown in Table 1). However, MCT^+^IL-17^+^ cells in the intratumor tissues did not correlate with any clinical characteristic assessed, including H. pylori infection status, tumor size, degree of differentiation and classification of TNM.

### Increased intratumor MCT^+^ cell, IL-17^+^ cell, and MCT^+^ IL-17^+^ cell densities were correlated with poor overall survival

**Figure 4:**
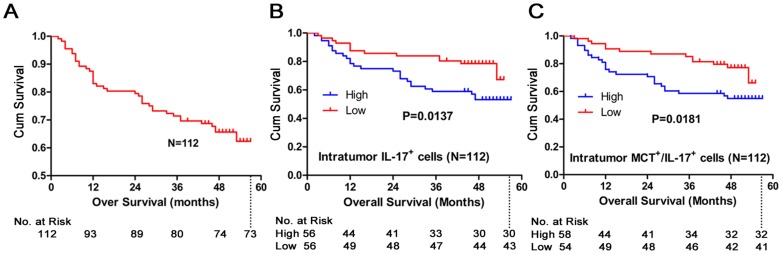
Intratumor accumulation of IL-17^+^ cells and MCT^+^IL-17^+^ cells predicts poor survival in gastric cancer patients. (A) The overall survival for all enrolled 112 patients (B) High intratumor IL-17^+^ cells infiltration conferred a significant high risk of death. (C) Patients with high MCT^+^IL-17^+^ cell intratumor had significant poorer survival than patients with low MCT^+^IL-17^+^ cell.

On univariate analysis (Table 3), the conventional clinicopathological features predictive of poor overall survival (OS) were poorly differentiated tumors, and advanced TNM stage. For further analysis, numbers of MCT^+^ cells, IL-17^+^ cells, and MCT^+^ IL-17^+^ cells were divided into two groups: above and below the median values. Kaplan-Meier survival curves were prepared using the log-rank test to further investigate the association with OS. Patients with high numbers of intratumor infiltrating MCT^+^ cells and IL-17^+^ cells had a poorer OS than those with lower numbers of MCT^+^ cells (p = 0.004) and IL-17^+^ cells (p = 0.014, Fig. 4B). Additionally, patients with higher numbers of MCT^+^ IL-17^+^ cells had shorter survival duration than those with lower numbers of MCT^+^ IL-17^+^ cells (p = 0.018, Fig. 4C).

**Table 3:** Univariate and Multivariate analysis of factors associated with overall survival in 112 gastric cancer patients.

*Variables*	Univariate b	Multivariate c		
	P-value	HR	95% CI	P-value
*Gender (Male vs. Female)*	0.753			NA
*Age (years)(>60 vs. ≤60)*	0.101			NA
*Tumor size (>5 cm vs. ≤5 cm)*	0.086			NS
*Tumor location*				
*(lower +middle +others vs. higher)*	0.112			NA
*Degree of differentiation*				
*(Poorly vs. well and moderately)*	0.017			NS
*TNM stage* a *(III vs. I+II)*	<0.001	3.832	1.679–8.745	0.001
*Intratumor positive (High vs. Low)*				
*MCT positive cells*	0.004	2.057	1.048–4.037	0.036
*IL-17 positive cells*	0.014			NS
*MCT/IL-17 double positive cells*	0.018			NS

HR hazard ratio; CI confidence interval; NA not applicable; NS not significant.

a 7^th^ Edition of American Joint of Committee On Cancer.

b Univariate analysis were performed by the Kaplan-Meier analysis model and log-rank test.

c Multivariate analysis were performed by Cox proportional hazards models with the backward likelihood stepwise procedures.

Variables of P<0.1 in univariate analyses were included in a multivariate Cox proportional hazards analysis, we found that both intratumor MCT-positive cells (HR  = 2.057; 95% CI: 1.048–4.037; p = 0.036) and TNM stage (HR  = 3.832; 95% CI: 1.679–8.745; p = 0.001) were independent prognostic factors (Table 3). Indicating that patients with higher numbers of intratumor MCT-positive cells were nearly 2.1-fold more likely to die than those with lower numbers of these cells.

### Immunohistochemical characteristics and the association between MCT^+^ IL-17^+^ cell density and immunochemical variables

**Figure 5:**
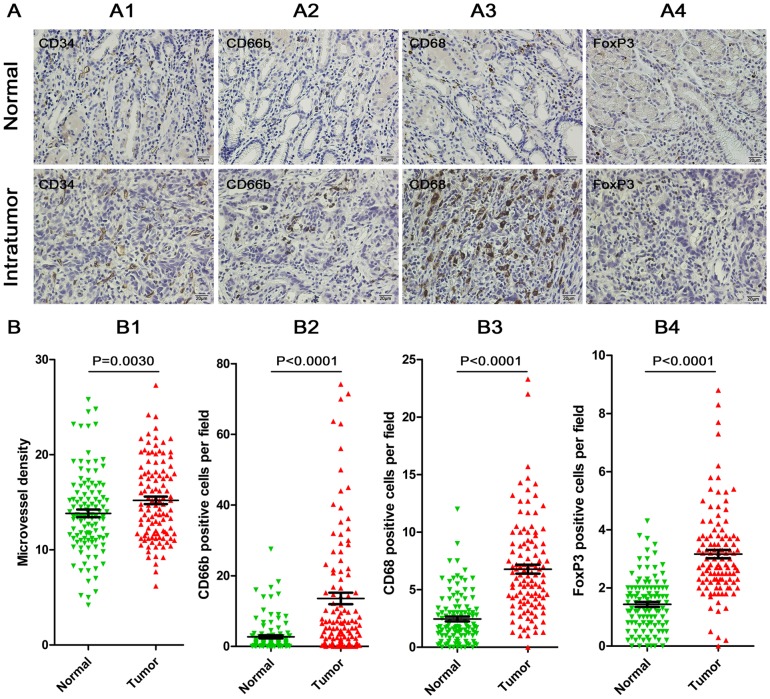
Distribution of immunostaining parameters in 112 gastric cancer patients. A. representative fields of CD34^+^ microvessels (A1), CD66b^+^ neutrophils (A2), CD68^+^ macrophages (A3) and FoxP3^+^ regulatory T lymphocytes (A4) in intratumor and normal tissue (brown, shown at 400× magnification). B. Absolute cell numbers of CD34^+^ microvessels (B1), CD66b^+^ neutrophils (B2), CD68^+^ macrophages (B3) and FoxP3^+^ lymphocytes (B4) per high-power field in intratumor and normal tissue were analyzed. The horizontal lines represent the mean ± standard error value of the group. P values were calculated by paired sample t-test for CD34^+^ microvessels and FoxP3^+^ lymphocytes, while Man-Whitney test was used for CD66b^+^ neutrophils and CD68^+^ macrophages analysis.

IL-17 is a known pro-inflammatory factor, which might promote tumor growth through fostering angiogenesis and recruiting neutrophils, as well as recruitment of other inflammatory and immune cell types. To explore the potential mechanism(s) of IL-17 accumulation in the tumor microenvironment with poor prognosis. We performed immunohistochemistry for various inflammatory mediators, especially, microvessel density, neutrophils, macrophages, and regulatory T cells in the entire study population (*n* = 112). Representative fields of CD34^+^ microvessels, CD66b^+^ neutrophils, CD68^+^ macrophages, and FoxP3^+^ regulatory T lymphocytes in intratumor and normal tissue are shown in Fig. 5A. The densities of intratumor CD34^+^ microvessels (p = 0.0030), CD66b^+^ neutrophils (p<0.0001), CD68^+^ macrophages (p<0.0001) and FoxP3^+^ regulatory T lymphocytes (p<0.0001) were significantly higher than those in normal tissues (Fig. 5B).

**Figure 6:**
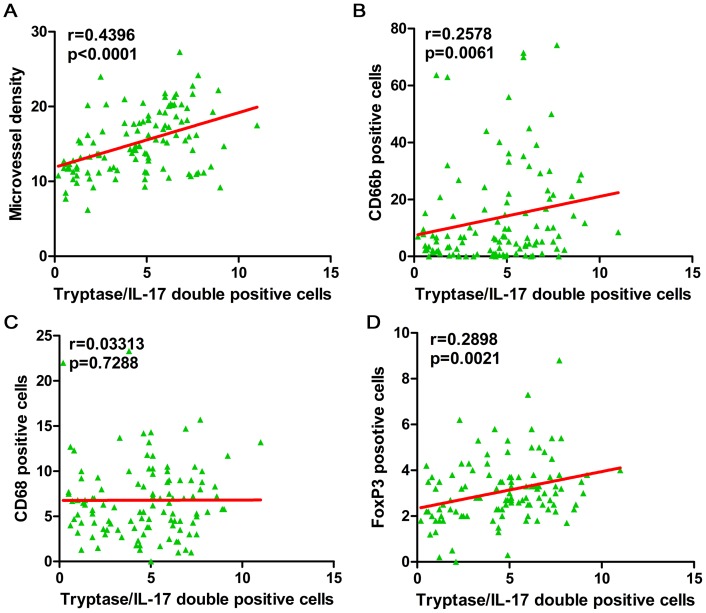
Correlation between tumor-infiltrated parameters with MCT^+^IL-17^+^ cells. (A) CD34^+^ microvessel density. (B) CD66b^+^ neutrophils. (C) CD68^+^ macrophages. (D) FoxP3^+^ lymphocytes. P values were determined by Pearson correlation coefficient for CD34^+^ microvessels and FoxP3^+^ lymphocytes, while Spearman correlation coefficient was used for CD66b^+^ neutrophils and CD68^+^ macrophages analysis.

Next, we analyzed the correlation between intratumor MVD, neutrophil density, macrophage density, regulatory T cell density, and the density of IL-17^+^ cells, particularly MCT^+^ IL-17^+^ cells. The results showed a significant correlation between MVD and IL-17^+^ cells (r = 0.4040, p = 0.0001), while significant, but weak, correlations were found between IL-17^+^ cells and neutrophils (r = 0.2135, p = 0.0238) and Tregs (r = 0.2963, p = 0.0017) (data not shown). However, we further analyzed the correlation between MCT^+^IL-17^+^ cells and those inflammatory mediators. A stronger significant correlation was found between MCT^+^ IL-17^+^ cells and MVD (r = 0.4396, p<0.0001). However, the correlations between MCT^+^ IL-17^+^ cells and neutrophils (r = 0.2578, p = 0.0061) and Tregs (r = 0.2898, p = 0.0021) were similar to that of IL-17^+^ cells (shown in Fig.6).

### Colocalization between IL-17R and various inflammatory and immune cells

**Figure 7:**
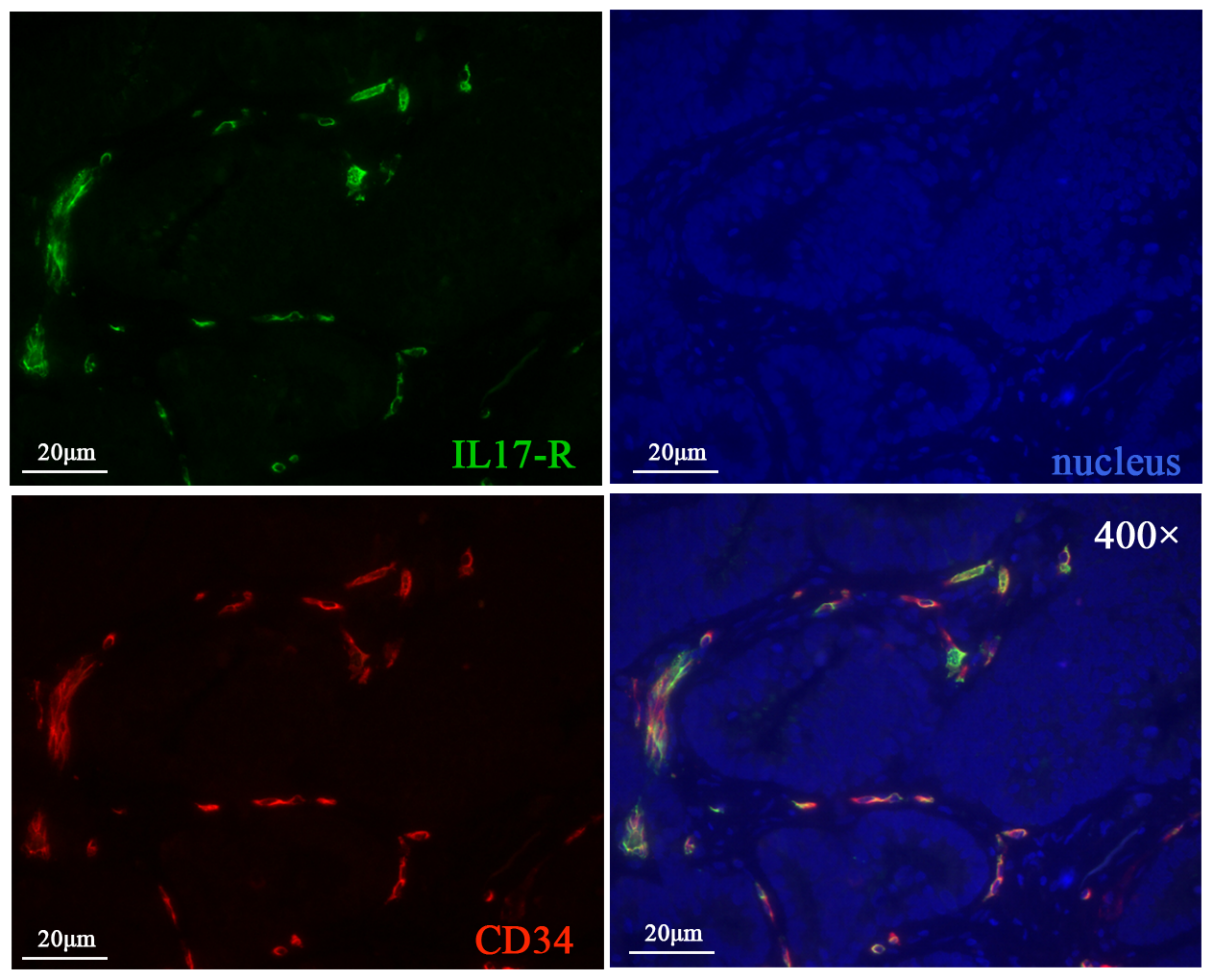
Colocalization between IL-17R (green) and CD34^+^ vascular endothelial cells (orange).

In order to explore the mechanism(s) under the closely relationship between IL-17 producing cells and MVD, as well as other inflammatory and immune cells. We performed colocalization experiments to evaluate the expression of IL-17R in vascular endothelial cells, neutrophils, macrophages and regulatory T cells (random selected, n = 20). Our results showed that the majority of vascular endothelial cells expressing IL-17R (6–58% of vascular endothelial cells; Fig.7) in intratumor tissues. Consistent with our hypothesis, barely neutrophils and regulatory T cells were detected expressing IL-17R. Interestingly, we found expression of IL-17R in the surface of tumor cells. (Fig. S2).

## Discussion

Immune escape plays a key role in the genesis and development of cancer. The mediators and cellular effectors of the immune system are important components of an immunosuppressive tumor environment. The role of interleukin-17, as a pro-inflammatory factor, has attracted attention recently in cancers, autoimmune diseases, and immunodeficiency diseases. CD4^+^ Th17 cells were long thought to be the sole cellular source of IL-17 in the human tumor microenvironment [23]. However, increasing evidence has demonstrated that various cell types can produce IL-17 [3], [24]. Mast cells, an important cellular component of the innate immune system, have been recognized as an important cellular source of IL-17. Indeed, they can produce and/or secrete a variety of cytokines and chemokines that contribute to immune defense and inflammation, including recruitment of leukocytes and vascular cell proliferation. Recently, it has been demonstrated that mast cells infiltrate into the tumor microenvironment via the SCF/c-kit signaling pathway, leading to exacerbation of inflammation and immunosuppression [25]. Mast-cell-derived IL-17 has been investigated in several autoimmune diseases, including rheumatoid arthritis [13], psoriasis [26], and spondylarthritis [27], in which mast cells were a major cellular source of IL-17, and showed a strong correlation with pathogenesis and disease progression. However, the source of the increased tumor-infiltrating IL-17 level and the contribution to the progression of the tumor, as well as the underlying mechanism(s), remained poorly understood.

In present study, we observed that IL-17-expressing cells in human gastric cancer samples exhibited two phenotypes. Some had a relatively regular phenotype, ovoid/plasmacytoid shapes, but some had more irregular shapes. Furthermore, we found that IL-17^+^ lymphocytes comprised only ∼10% of the IL-17-expressing cells, in contrast with previous studies in human tumor microenvironments [7], [23]. Recently, Bo Wang et al. [28] found that mast cells, but not T cells or macrophages, were the predominant type expressing IL-17 in esophageal squamous cell carcinoma tissues. Interestingly, we also found that the majority of IL-17^+^ cells colocalized with MCT^+^ cells (14–68.1% of IL-17^+^ cells), indicating that mast cells were the predominant cellular source of IL-17 in human gastric cancers. Several studies have shown that IL-17 impairs immune surveillance and promotes angiogenesis and carcinogenesis in tumors [1], [29], suggesting that the inflammation environment driven by levels of endogenous IL-17 may contribute to tumor progression. Our findings suggested that the level of IL-17-expressing cells was higher in tumor tissue samples than the corresponding normal tissue samples and that mast-cell-derived IL-17 was more abundant in human gastric cancers. Kaplan-Meier analysis revealed that intratumoral IL-17^+^ cells and intratumoral MCT^+^ IL-17^+^ cells were correlated with worse survival in human gastric cancer patients.

The precise mechanism(s) underlying the association between increasing levels of IL-17 in the tumor microenvironment and tumor progression remain(s) unclear. A study reported that IL-17 promote tumor development through the recruitment of myeloid derived suppressor cells (MDSCs), such as CD11b^+^Gr1^+^ cells, to the tumor environment [30]. Meanwhile, several studies have demonstrated that IL-17 can enhance the growth of vascular endothelial cells and influence the angiogenic progress by increasing the secretion of cytokines, such as TNF-α, IL-8, and VEGF [29], [31], including a study that reported IL-17-mediated paracrine network promotes tumor resistance to anti-angiogenic therapy [32]. Consistent with this, our results indicated a significant correlation between MVD and IL-17^+^ cells. It has been well established that mast cells could stimulate vascular proliferation [33], in our study, we found a more significant correlation between MVD and MCT^+^ IL-17^+^ cells, demonstrating that these IL-17 producing mast cells have stronger effect on promoting angiogenesis. In addition, we found that the majority of vascular endothelial cells expressing IL-17R, more interestingly, we found that gastric cancer cells were positive for IL-17R. Previous studies have reported that IL-17R expression was associated with tumor malignancy [34], [35]. Combining these results, IL-17 infiltration may have promoted tumor progression by enhancing angiogenesis in the tumor microenvironment through the axis of IL-17/IL-17R, function on vascular endothelial cells and tumor cells. Another possible mechanism lies in the recruitment of inflammatory cells by IL-17, such as neutrophils and macrophages. It has been demonstrated that IL-17 promotes tumor progression through direct effects on the recruitment of neutrophils [36], [37]. In our study, intratumoral IL-17^+^ cells and MCT^+^ IL-17^+^ cells both showed weak, but significant, correlations with the number of intratumor neutrophils, while intratumor macrophages showed no significance. Although, barely neutrophils were found expressing IL-17R. Mast-derived IL-17 might play a key role in the recruitment of neutrophil by CXC chemokines, such as CCL2, as described previously [38]. Evidence from various cancers demonstrates that the proportion of Regulatory T cells (Tregs) is increased in tumor tissue in patients with multiple malignancies [39], [40]. It has been reported that Tregs recruitment had a key role in establishing a VEGF-rich tumor microenvironment and increasing tumor angiogenesis [41]. Previous study has reported the regulatory role of mast cells in that they interact with conventional CD4^+^ T cells to generate IL-10 producing regulatory T cells through the axis of ICOSL/ICOS [42]. Ganeshan et al. [43] reported that Tregs actually enhance, rather than inhibit, mast cell production of IL-6, a pleiotropic cytokine, which has been shown to play a pivotal role in the regulation of the balance between IL-17-producing cells and FoxP3^+^ T regulatory cells. Thus, intense correlations may exist between Tregs and IL-17-producing mast cells. In our study, the correlation between IL-17^+^ cells and Tregs was weak, but significant, as well as the MCT^+^ IL-17^+^ cells. These results demonstrated that IL-17 producing mast cells might induce the differentiation and accumulation of Tregs by secreting various of cytokines and chemokines, which need to be further elucidated.

To our knowledge, this is the first report of the phenotype and distribution of intratumor IL-17-producing cells and their clinical relevance, and particularly the prognostic value of intratumor mast cell-derived IL-17 in gastric cancer. Our data demonstrate that most IL-17-producing cells were mast cells, whereas IL-17^+^ lymphocytes were rare. Furthermore, we found that intratumoral IL-17^+^ cells and intratumoral MCT^+^ IL-17^+^ cells were correlated with worse survival. However, the mechanism(s) of mast cell infiltration into the tumor microenvironment and the specific mechanism between the overexpressing IL-17 and the inflammatory and immune cells remain largely unknown. Furthermore, the distinctive function of stored and secretory IL-17 still needed to be illustrated. Thus, prospective studies focusing on mast cell recruitment and IL-17 production in the stomach tumor microenvironment are necessary to assess the predictive value of intratumor mast-cell-derived IL-17 further.

## Supporting Information

Figure S1 **Colocalization between c-kit and mast cell tryptase (MCT).**
(TIF)

Figure S2 **Colocalization between IL-17R (green) and neutrophils, macrophages, regulatory T cells (orange).**
(TIF)
