# Supplementary figures and images for: Intratumor IL-17-Positive Mast Cells Are the Major Source of the IL-17 That Is Predictive of Survival in Gastric Cancer Patients
Source: PLoS One. 2014 Sep 8;9(9):e106834. doi: 10.1371/journal.pone.0106834 (PMC4157802; doi:10.1371/journal.pone.0106834)

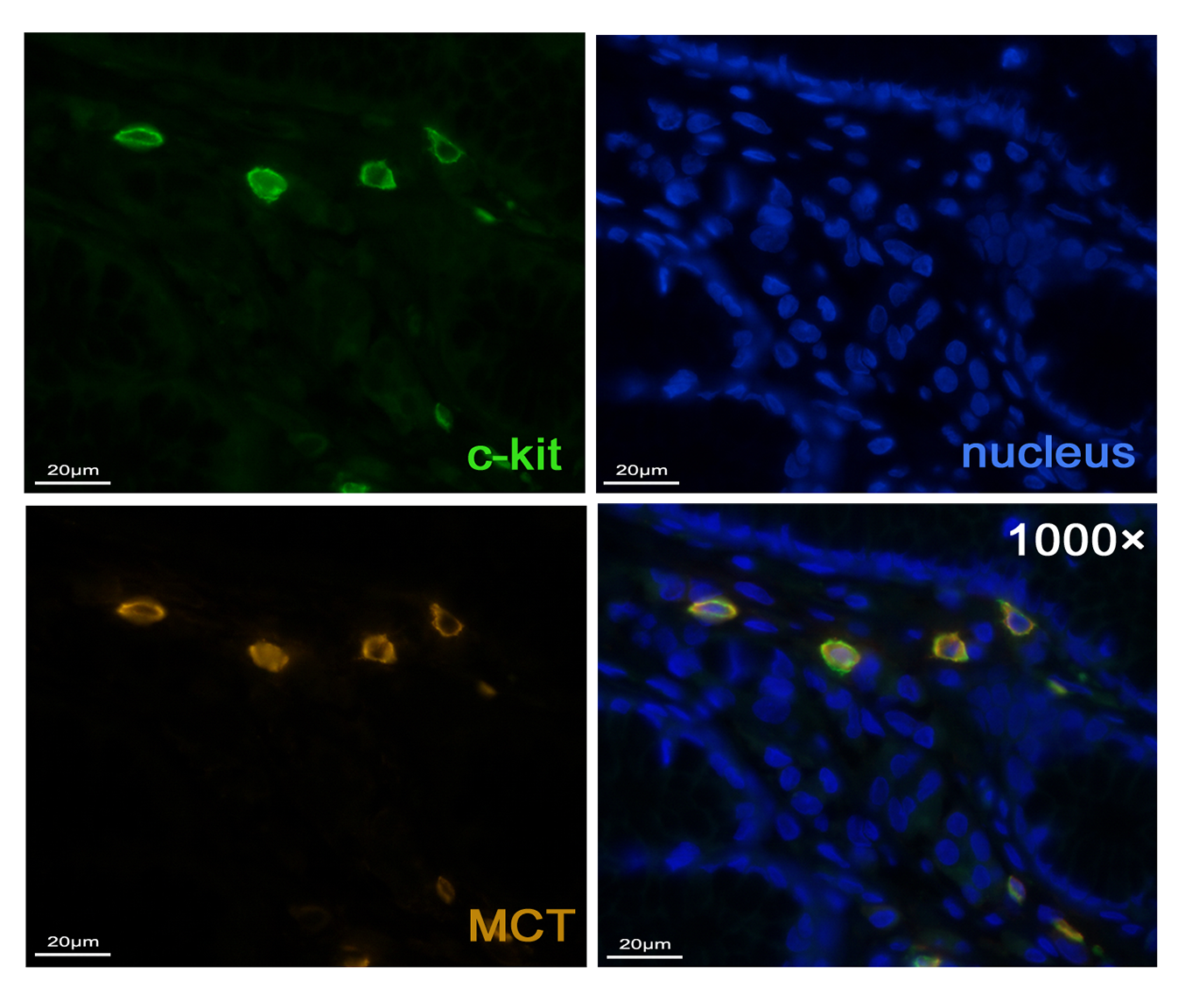

Supplement: Figure S1 — Colocalization between c-kit and mast cell tryptase (MCT). (TIF) [file pone.0106834.s001.tif]

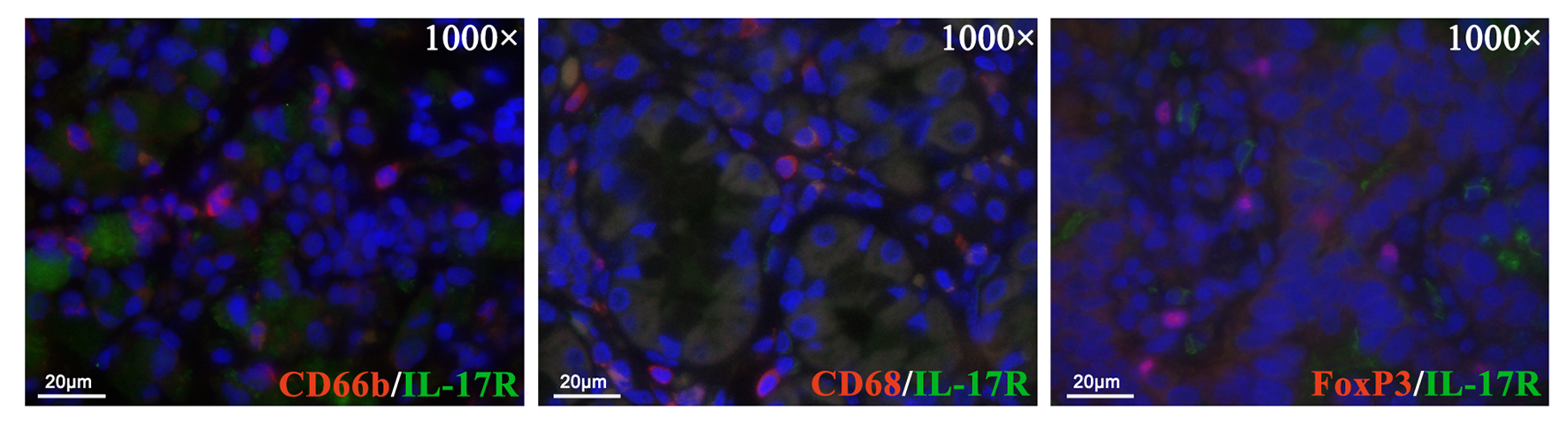

Supplement: Figure S2 — Colocalization between IL-17R (green) and neutrophils, macrophages, regulatory T cells (orange). (TIF) [file pone.0106834.s002.tif]
